# Early warning signs for saddle-escape transitions in complex networks

**DOI:** 10.1038/srep13190

**Published:** 2015-08-21

**Authors:** Christian Kuehn, Gerd Zschaler, Thilo Gross

**Affiliations:** 1 Vienna University of Technology, 1040 Vienna, Austria; 2 TNG Technology Consulting, 85774 Unterföhring, Germany; 3 University of Bristol, Merchant Venturers School of Engineering, BS8 1TR Bristol, UK

## Abstract

Many real world systems are at risk of undergoing critical transitions, leading to sudden qualitative and sometimes irreversible regime shifts. The development of early warning signals is recognized as a major challenge. Recent progress builds on a mathematical framework in which a real-world system is described by a low-dimensional equation system with a small number of key variables, where the critical transition often corresponds to a bifurcation. Here we show that in high-dimensional systems, containing many variables, we frequently encounter an additional non-bifurcative saddle-type mechanism leading to critical transitions. This generic class of transitions has been missed in the search for early-warnings up to now. In fact, the saddle-type mechanism also applies to low-dimensional systems with saddle-dynamics. Near a saddle a system moves slowly and the state may be perceived as stable over substantial time periods. We develop an early warning sign for the saddle-type transition. We illustrate our results in two network models and epidemiological data. This work thus establishes a connection from critical transitions to networks and an early warning sign for a new type of critical transition. In complex models and big data we anticipate that saddle-transitions will be encountered frequently in the future.

The low-dimensional systems that are commonly investigated in the context of critical transitions (or tipping points)^1^,^2^ typically settle to stable, but not necessarily stationary, states^3^. The defining features of such states is that the system returns exponentially to the state after sufficiently small perturbations^4^. When environmental parameters change, a critical transition may occur when thresholds are crossed, where the system becomes unstable to certain perturbations. Low dimensional systems then typically depart quickly from the original state before approaching another, possibly distant, state. The thresholds at which such transitions occur are called *bifurcation points*^5^ and the corresponding transitions are *local bifurcations*^3^,^6^. Warning signs for such bifurcation-induced transitions detect the loss of exponential restoring dynamics either through its impact on the statistics of noise-induced fluctuations^1^,^7^,^8^ or by direct measurement of recovery rates^9^,^10^. Thus, bifurcation-induced critical transitions are well understood, and the corresponding warning signs have been analyzed mathematically^11^ and tested in experiments^12^,^13^.

For understanding the alternative mechanism of critical transitions that is the focus of the present paper, consider that many real-world complex systems do not reside in stable states. An intuitive example is provided by the outbreak of an epidemic in a population occuring under stable enviromental conditions. Although the precise mechanism for spreading after invasion can be very complex^14^, many examples show that introducing a certain new or previously extinct pathogen into an unprepared population can have drastic consequences. This illustrates that the original state of the system, in which the pathogen is absent, was unstable with respect to a specific perturbation, corresponding to the introduction of the pathogen. Note that even after its introduction, the pathogen may be extremely rare for an extended amount of time, residing in small subpopulations or animal vectors, such that on a macroscopic level a disease-free state is still observed for significant time. We may only see a very small rise in the number of infected individuals for a long time under fixed environmental conditions but eventually a drastic jump to an endemic regime occurs (see Supplementary Information, Section 2, for a simple compartmental epidemic model illustrating this effect).

The example above differs fundamentally from bifurcation-induced transitions, because the qualitative change is not induced by a change of environmental parameters, but rather by a specific ‘rare’ perturbation. A system is susceptible to such perturbation-induced transitions if it resides in a saddle point, a state that is stable with respect to some perturbations, but unstable with respect to others. In the following we refer to critical transitions caused by the departure from saddle points far from bifurcations as *saddle-escape transitions*; see also (Fig. 1) for a mathematical normal form example for passage near a saddle; we remark that this example is generic in the sense that mathematical theory guarantees that other systems with nondegenerate saddles show the same dynamics up to coordinate changes and by using a suitable notion of equivalence for the dynamics^4^.

In simple low-dimensional, modular or symmetric (i.e. effective few-variable) systems that are typically studied in the context of early-warnings signs, saddle-escape transitions may occur^15^,^16^ but are relatively rare in practice, particularly in comparison to saddles in high-dimensional (many-variable) systems. To understand this difference, first, consider the abundance of available saddle states. Whether a steady state is an attractor, repeller, or saddle is determined by the eigenvalues of the systems Jacobian matrix, which provides a linearization of the system around the state in question^4^,^5^. A state is a repeller when all of these eigenvalues have positive real parts, an attractor if all eigenvalues have negative real parts, and a saddle when there are eigenvalues with positive real part and also eigenvalues with negative real-part. In a complex heterogeneous system the eigenvalues can, to first approximation, be considered as random variables^17^ that have negative real parts with a certain probability *q*. Since the total number of eigenvalues increases with the number of variables *N*, the proportion of attractors decreases as *q*^*N*^, the proportion of repellers decreases as (1 − *q*)^*N*^, whereas the proportion of saddles increases rapidly with increasing system size (see Supplementary Information, Section 1). We can therefore expect to find an abundance of saddle states in generic complex high-dimensional heterogeneous systems.

Given the existence of saddles we may also want to to ask whether systems can even approach a single saddle. This is possible, e.g., if a trajectory exists that connects the saddle state to itself or several trajectories connect between different saddles. Such *homoclinic* or *heteroclinic orbits*^4^,^5^ exist in low-dimensional systems only if certain conditions are met exactly, e.g. parameters are tuned exactly right. However, if we allow parameters to change dynamically, the dimension of the system increases naturally and long-time homoclinic and heteroclinic dynamics can appear robustly. A simple example is the fold-homoclinic (or square-wave) bursting mechanism observed in many neurons^18^, where additional macroscopic slow gating variables are frequently introduced to capture the complex processes in the neuron in addition to the usual voltage dynamics. This leads to a robust repeated passage near a homoclinic structure. Furthermore, if there are certain symmetries in the system, then heteroclinic behavior may occur generically as well^19^,^20^.

Saddle-escape can only be considered a critical transition if the system resides in the saddle for a sufficiently long time in comparison to the dynamics away from the saddle. In this case, the saddle is perceived as the natural state. The residence time of a system near a saddle can be long as the system moves slowly near steady states (see Supplementary Information, Section 1, for a standard calculation of the residence time). When the system is subject to a small perturbation, its response can be decomposed into different fundamental modes. Some of these modes decay quickly, restoring the system to the steady state. However, at least one mode exists that once excited initially grows exponentially, and thus leads to an escape from the saddle. Here we consider the state where the initial perturbations from those growing modes are very small or entirely absent, since otherwise the system would depart very quickly from the saddle.

In full mathematical generality we would usually expect that a typical perturbation of the system excites all modes to some degree, and thus triggers departure from the saddle point. Based on this reasoning we would expect that already the first perturbation of a system residing in a saddle launches it into exponential departure. However, in real high-dimensional systems the situation is not so simple. For example, hidden symmetries or constraints may exist that effectively decouple certain variables from perturbation of other variables. The most important constraint is the absolute nature of the zero line. Consider again the example of a novel pathogen introduced into a population. Here it is immediately apparent that a perturbation cannot excite the exponential growth of the infected population, unless it already involves the introduction of at least some infected individuals. Furthermore, the perturbation direction to the saddle may initially be very weak in comparison to the strong stable directions, which also leads to long residence times near saddle points (see Supplementary Information, Section 2, for a simple compartmental epidemic model illustrating this effect). Finally, in many models stochastic effects cannot be ignored close to the steady state (see Supplementary Information, Section 2). For instance it is well known that small populations that are feasible in the deterministic limit may still go extinct due to fluctuations in populations size, a phenomenon known as demographic extinction^21^. These mechanisms, i.e., micro-level stochastic extinction, slowness of departure and decoupling of perturbation modes, can effectively stabilize the saddle state for a long time until a particular perturbation, or series of perturbations, launches the system on an escaping trajectory. Considered together, the well known-arguments presented above suggest that saddle-escape may play a role as a critical transition, particularly in high-dimensional systems.

Let us now ask whether warning signs for saddle transitions can exist. Because saddle-escape is triggered by the occurrence of a certain perturbation, which is inherently unpredictable in the context of the model, we cannot hope to detect the saddle escape far before the exponential departure from the saddle starts. However, consider that in contrast to bifurcation-induced transitions, saddle escape will generally occur at states that are far from bifurcations. Such states are said to be *hyperbolic*, and perturbations to these states grow or decline exponentially depending upon the perturbation direction. The departure form the saddle is initially slow and dynamics around the saddle are often perceived as meta-stable. Phenomenologically, the transition therefore appears very similar to a bifurcation-induced critical transition, showing first a slow drift, followed by a sharp spike. Arguably, in many applications it should thus be possible to restore the system to the saddle during the initial phase where the intrinsic dynamics are still slow. Our aim is thus to detect the saddle-escape after the critical perturbation has occurred, but before the system has moved so far from the saddle that the dynamics has accelerated too much. In fact, if we know, a priori, the system is approaching a saddle, not just a fully stable state, then the warning sign described below may help to detect a potential critical transition already at the very beginning of the metastable phase; otherwise, we can apply it during the metastable phase.

In our mathematical treatment we consider a scenario where the system starts from some initial point, then approaches the saddle and stays for a significant time in the vicinity of the saddle point before departing again. For detecting the onset of the departure we exploit the exponential form of the departure trajectory. Between two time points *t*_1_ and *t*_2_ the logarithmic distance along a trajectory close to the saddle point x^*^ will eventually be dominated by a scaling of the form


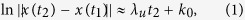


where *λ*_*u*_ > 0 is the real part of the largest eigenvalue of the Jacobian and *k*_0_ is a constant (see Supplementary Information, Section 2), i.e., the logarithmic distance increases linearly in forward time. Although saddles generically have positive eigenvalues, the influence of *λ*_*u*_, which can be estimated by Eq. (1), on the overall system dynamics is small as long as the system is approaching the saddle sufficiently close to its stable directions. In this case, the dynamics does not yet involve a significant component in the direction of unstable eigenvectors or the system would not approach the saddle at all.

Let us illustrate this again by the epidemics example. While the pathogen is absent the variable that captures the density of the infected population remains fixed to zero, since there are no dynamics in the infected population variable we only measure a stable eigenvalue with real part *λ*_*s*_ < 0 via an analogous logarithmic scaling relation as Eq. (1) (see Supplementary Information, Section 2), where the logarithmic distance decays linearly in forward time. Only after the pathogen is introduced and the infected population starts growing, we start to pick up the positive eigenvalue *λ*_*u*_ associated to the exponential growth.

Hence, we may use logarithmic distances between points as a measure to determine, which eigenvalue *λ* is currently dominating as shown in (Fig. 1(b,c)). If we find negative *λ* this signals that we are in a regime dominated by a stable direction and approach the saddle, whereas a positive *λ* signals a departure. Therefore, the emergence of positive *λ* beyond a certain threshold can provide a warning signal for saddle-escape transitions. If we know, a priori, that we are approaching a saddle, not just a fully stable state, then the warning sign already detects the saddle when the logarithmic distance reduction decays linearly. If it could be a stable or saddle state, we can only apply the logarithmic distance reduction warning sign during the metastable phase near the saddle.

To illustrate the saddle-escape mechanism for critical transitions and to test the proposed early-warning signal we consider two recent adaptive-network models in which critical transitions as well as saddle-type behavior play key roles in the dynamics and an epidemiological data set. The first system is a model from evolutionary game theory, in which the evolution of cooperative behavior in a network of interacting, self-interested agents is studied^22^.

The model describes a network of agents, connected by social contacts. Each agent pursues one of two possible strategies, which we call cooperate and defect. The agents engage in pairwise interactions with their neighbors, which are modeled as a snowdrift game^23^. In this game the highest social payoff in produced by mutual cooperation. However, for the individual agent, defecting yields a higher payoff when the agent is interacting with a cooperator. In time both the agents’ strategies and the network of interactions change as agents switch to the strategy that performs optimally in the population, and also rewire their connections to other agents following the more successful strategy (see Supplementary Information, Section 3).

Previous work^22^ has shown that in the limit of infinite population size the system robustly approaches the state of full cooperation, where the probability that a randomly drawn agent follows the cooperative strategy is one. In large, but finite, networks a state of almost full cooperation is reached that is disrupted by large outbreaks of defection (see Supplementary Information, Section 3).

We now explore whether the logarithmic scaling discussed above is capable of providing early-warning of these outbreaks. We build on full agent-based simulation but our analysis focuses on a pair of observables. In particular, we study the density of cooperators, *x*_1_, i.e., the proportion of agents whose current strategy is cooperation, and the density of links (per agent) that exist between cooperators and defectors, *x*_2_. Note that per-capita densities of populations are also frequently the only natural variables available in data, so they are a natural choice to detect critical transitions and warning signs.

First, let us establish that the outbreaks of defection are indeed triggered by a saddle-escape transition. Although bifurcation-induced transitions are not the main focus of this paper, we provide a brief description of the known bifurcation structure here as a parameter is varied and consider the dynamics as a function of the rewiring *p*, which measures the relative time scale of structural changes of the network to internal changes in the agents strategy. This rewiring rate was previously identified as a key parameter of the system (see also Supplementary Information, Section 3).

Increasing *p* at a constant rate from a small initial value, we observe two main dynamical changes. First, a stationary solution turns into stable oscillations, which increase in amplitude and period. Here we observe the classical increase in variance before a Hopf bifurcation^1^,^11^ (Fig. 2(b1)), which is a good predictor for the transition from random fluctuations to small deterministic oscillations. Second, critical transitions occur at larger rewiring rates, where the cooperator density *x*_1_(*t*) drops sharply to lower values before rising again slowly. These transitions are associated to the presence of a homoclinic loop in the system^22^, which is attached to the fully cooperative state 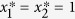.

In (Fig. 2(b2)), we show that the period *T* of the oscillations may grow rapidly in finite time, indicating a global bifurcation that gives rise to the homoclinic loop (see Ref. 5 for further background on homoclinic bifurcations). Trajectories close to the homoclinic loop remain near the saddle point for a long time before making a fast excursion. Locally, near 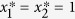, this is precisely the situation of saddle-escapes. The logarithmic distances *d*_1,2_ are shown in (Fig. 2(b3)). As a warning sign to predict the rapid drops in *x*_1_ in the range of *p* ∈ [0.93, 0.96], we assume that we know an eventual saddle-instability will happen so we just have to look for a change of linear scaling induced by stable directions for *d*_1_ during the phases when *x*_1_ is gradually increasing. These changes can be seen for *p* ≈ 0.944 and *p* ≈ 0.957 as predicted by the theory see (Fig. 2 (b3)). Note that the complex saddle-escape dynamics occurs in the regime of relatively large re-wiring rate, when the dynamical process of node update and re-wiring act on similar time scales, while the bifurcation-induced warning signs worked for low re-wiring in a quasi-stationary scenario.

As a second example, we consider a susceptible-infectious-susceptible (SIS) epidemiological model on an adaptive network^24^,^25^. The network consists of susceptible (S) and infectious (I) agents. An infection spreads along S-I-links with probability *p* per unit time, infectious agents recover with probability *r*, and susceptible agents try to avoid infectious ones by rewiring S-I-links to S-S-links with a probability that is proportional to the total number of infectious agents (see Supplementary Information, Section 4).

In simulations of this system the number of infectious agents shows distinguished peaks in time, which can be interpreted as epidemic outbreaks (Fig. 3). In (Fig. 3(b)) the logarithmic distances between consecutive points are shown for the density of I-nodes and S-I-links. Despite the strong fluctuations away from the peaks, both warning signals show three phases after a peak: (1) strong stabilization, (2) plateau- or noise-type behavior and (3) a trend towards instability before the next spike (see Fig. 3(a2)). In fact, similar phases can also be observed for the first model in (Fig. 2(b3)).

Furthermore, we note that the logarithmic distance increases much earlier for the S-I-links than for the I-nodes, so that monitoring the links between infectious and susceptible agents provides an earlier warning signal for epidemic outbreaks (Fig. 3). This is in accordance with the intuitive idea that knowledge about the contact dynamics among infectious and susceptible agents should allow to predict epidemic outbreaks more easily.

The two models suggest that there are regions in parameter space where saddle-escapes play a key role. However, it is also known that epidemic network models can exhibit bifurcation-induced critical transitions with classical warning signs critical transitions^11^,^26^. However, in those cases one usually assumes that a parameter, e.g. the infection rate, is very slowly varying and this causes the critical transition. This directly motivates the question whether there are data sets available where epidemics are driven by saddle escapes.

Here we focus on repeated measles outbreaks documented biweekly between 1944 and 1966 in 60 cities in the United Kingdom^27^,^28^. The time series of the proportion of infected individuals shows long repeated periods of low disease prevalence interspersed with large, but very short, outbreaks. This strongly indicates that a saddle-type mechanism may be at work (Fig. 4(a)). We use a receiver-operating-characteristic (ROC) curve^29^,^30^ to quantify the performance of our warning sign for saddle escapes. We briefly recall, how ROC curves are calculated. First, one defines a scalar precursory variable *X* to be computed from observations before the transitions and considers a threshold *δ* such that if *X* > *δ* an alarm is given. Then the two ratios of correct alarms to the total number of actual tipping events and false predictions to the total number of non-tipping events are calculated for various thresholds *δ*. This provides a quantitative indicator for the ability of the precursor variable to detect tipping points (see Supplementary Information, Section 6, for more background on ROC curves and their interpretation).

For the ROC curves in our case, we compute a least squares fit of the parameter *λ*_*u*_ = :*X* from equation (1) within a time window of *k* data points as a precursory variable. We give an alarm for an imminent outbreak at the end of the time window when *λ*_*u*_ > *δ* for some threshold *δ*. This prediction is considered correct if the disease prevalence exceeds 0.1 within the next 5 data points, which corresponds to an outbreak of at least ten percent of the maximum outbreak coming up within the next 2.5 months. In (Fig. 4(b)) we show two ROC curves relating the rate of correct predictions to the rate of false positives for different threshold values *δ*. Both curves lie above the diagonal, indicating that our method is better than a purely random prediction. Moreover, the predictions using positive threshold values *δ* are, on average, better than the ones using *δ* < 0, as the former produce a ROC curve farther away from the diagonal. This is a natural result, because *δ* > 0 corresponds to the detection of an actual instability while 0 > *δ* ≫ −1 only corresponds to the detection of a weakly stable direction. Clearly, very short prediction times (small *k*) are problematic as they increase the error in *λ*_*u*_ leading to false alarms. Long prediction times (large *k*) lead to very few correct predictions because the exponentially stable approach to the saddle strongly dominates on long time scales. Although the ROC results show that we can potentially improve the prediction of epidemic outbreaks, the situation is far from ideal. The ROC curve is still quite far from the top-left corner in (Fig. 4(b)), which would be perfect prediction. Hence, there is substantial work to be carried out to try to increase the practical performance of the warning sign.

Note that, although we demonstrated many factors which strongly indicate a saddle-escape mechanism in measles data, it is unlikely that a test exists which can guarantee detailed knowledge of the underlying dynamical mechanisms based on just a relatively short uni-variate time series of the epidemic. However, there is evidence from several epidemic models, which do display saddle states^31^,^32^. It then remains as an open problem to link our warning sign analysis of measles data to particular models of measles^33^,^34^, which we leave as a challenge for future work. Furthermore, we remark that although the indicator we have used seems reasonably efficient, its robustness will strongly depend on the noise level in the system.

As a general comment, we note that the exact mechanism by which a critical transition occurs may well lie in the eye of the beholder. A given critical transition observed in nature may appear as a bifurcation-induced transition in one model and as a saddle-escape in a different model describing the same phenomenon with different variables.

In summary, we have identified a new type of critical transitions that could be relevant for real-world complex many-variable saddle-type systems. We proposed a simple, intuitive early warning signal for these transitions, and demonstrated the application of this warning signal in two adaptive network models and real-world data. We believe that this warning signal will be useful for detecting saddle-escape transitions in network models and data sets, for instance for ecological and socio-economic systems. Because the underlying mechanism considered here differs from the ones typically studied in critical transitions, the proposed early warning sign is complementary to existing approaches.

It is crucial to point out that significant additional research is necessary to make warning signs, for saddle-escape as well as the classical bifurcation-induced tipping scenarios, more applicable for many real-world applications. The first main issue is to connect results to a more detailed statistical analysis, which is currently work in progress by several groups^30^. A second challenge is to link qualitative modelling results better to quantitative warning signs. Indeed, both necessarily depend on each other for saddle- and bifurcation-mechanisms. In both cases the predictions are tremendously improved if one knows *a priori* that the current dynamical evolution of the system may be towards a destabilizing tipping scenario and one just does not know *where* the system tips, on what *time horizon* it happens, and which precise *mechanism* occurs. A third challenge is to keep new potential applications in perspective and there has been significant recent progress in this direction in realistic systems for bifurcation-induced transitions, for example in ecology^10^. Various models in neuroscience^18^ and in ecology^16^ suggest that saddle points can occur, which are bound to be preceeded by the generic logarithmic distance reduction we use as a warning sign. In an ecological context a natural future test case could be bio-invasions^35^,^36^,^37^,^38^.

## Additional Information

**How to cite this article**: Kuehn, C. *et al.* Early warning signs for saddle-escape transitions in complex networks. *Sci. Rep.* **5**, 13190; doi: 10.1038/srep13190 (2015).

## Supplementary Material

Supplementary Information

## Figures and Tables

**Figure 1:**
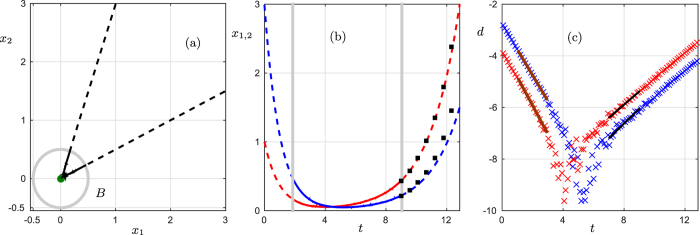
Dynamics near a planar saddle with small noise. (**a**) Phase space (*x*_1_, *x*_2_) with a trajectory (black) passing near the saddle point. The stationary state (dark green dot) and a circle (gray) of radius *r* = 0.5 indicate a neighborhood of the stationary state outside of which the trajectory is shown as a dashed curve. (**b**) Time series for *x*_1_ (red) and *x*_2_ (blue). The gray vertical lines indicate entry and exit to the ball 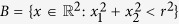. The black squares are predicted values from the warning signals obtained inside *B*. (**c**) Plot of the logarithmic distance reduction *d*(*T*) as crosses; (see Supplementary Information, Section 2). The red/blue linear interpolants yield two approximations for the stable eigenvalue *λ*_*s*_ ≈ −1.10, −0.99 and the black lines for the important unstable eigenvalue *λ*_*u*_ ≈ 0.51, 0.57; the true values are (*λ*_*s*_, *λ*_*u*_) = (−1, 0.5). The black squares in (**b**) can be obtained from 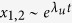. Note that the choice of *B* is a choice of sliding window length (or lead time) for prediction as in the case for bifurcation-induced tipping.

**Figure 2:**
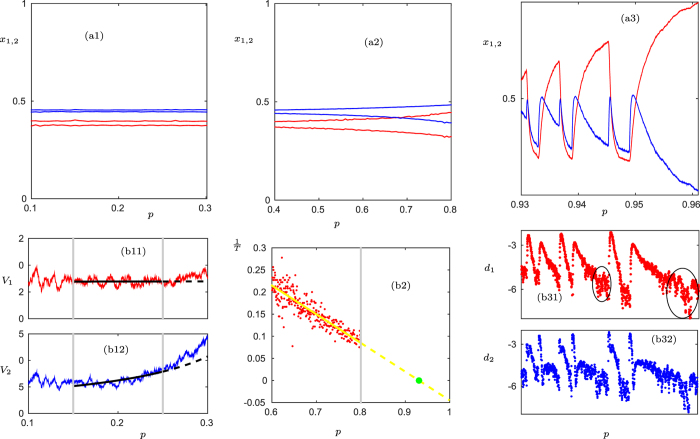
Critical transitions for an evolutionary game. (**a**) Time series for the density of cooperators *x*_1_ (red) and the density of defectors *x*_2_ (blue); note that we slowly increase the parameter *p* in time at a constant rate, i.e., *p* can be viewed as a time variable. In (a1)–(a2) the minima and maxima of a moving average are shown whereas (a3) shows the actual time series. The vertical dashed curve (thin black) in (a1) indicates the theoretically-predicted transition to oscillations; see (Supplementary Information, Section 3). In (b1) the variances for *x*_1,2_ are calculated using a moving window technique up to the gray vertical lines; note that the scaling of the *V*_1,2_-axis is 10^−5^. Observe that *x*_1_ does not show a clear scaling law while the scaling of *x*_2_ can be used for predicting the transition from steady state to oscillations using classical variance-based warning signs. The predicted transition point from extrapolating the increasing variance scaling law^11^ is marked as vertical dashed line (black) in (a2); note that there is a delay in the Hopf bifurcation point so the predicted critical transition to matches, from a practical viewpoint, the data better than the second-order moment closure theory^22^. In (b2) the period *T* of the oscillation is measured and 1/*T* is linearly interpolated to approximate the period blow-up^5^ point (yellow). This is used to predict the transition point (green) from a periodic to a saddle-type/homoclinic regime; note that this period blow-up is not the saddle-mechanism we focus on in this paper but another new warning sign we just note as an interesting related result. The predicted transition is marked by the dashed vertical line (green) in (a3). Then we also show the logarithmic distance reduction measured from (a3) in (b3). The ellipses in (b31) indicate the regime where the decay-scaling for the saddle-approach breaks down. Note that the ellipses are there to guide the eye. If one would want to give an explicit warning sign, a threshold for *λ*_*u*_ has to be specified, which is not done in this qualitative example. A detailed quantitative analysis of thresholds is carried out for a data set below using ROC analysis. Here we just want to point out the existence of saddles and the qualitative change in the distance reduction near the saddle, i.e. the parts (a3) and (b3) illustrate the main ideas for saddle-escape warning signs.

**Figure 3:**
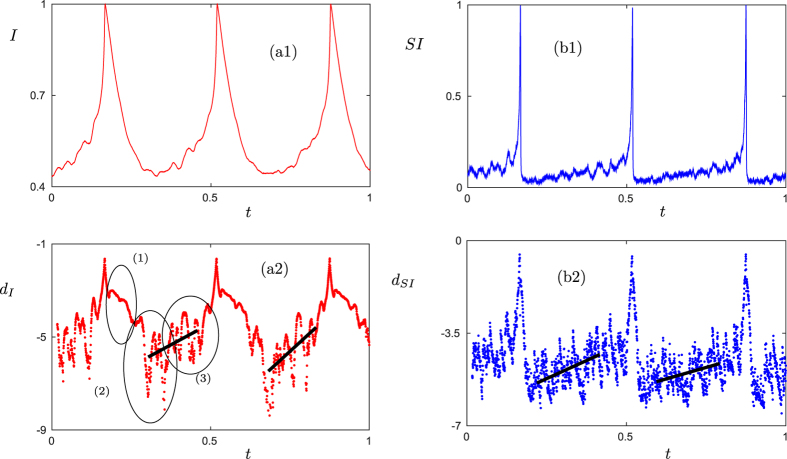
Epidemic outbreaks and prediction in an adaptive SIS model. (**a**) Normalized time series for the infected density *I* (red) and the susceptible-infected link density *SI* (blue). (**b**) Logarithmic distances for *I* and *SI* are shown as well (see Supplementary Information, Section 2). The linear interpolations (black) indicate the expected linear upward trend before a saddle-escape; the slopes of the four black lines (from left to right) are approximately 7.364, 12.516, 5.466 and 3.461 respectively. The three ellipses in (a2) highlight the three typical regimes between spikes discussed in the text and are there to guide the eye as in (Fig. 2). Parameter values for this figure are *p* = 0.0058, *r* = 0.002 and *w*_0_ = 0.6.

**Figure 4:**
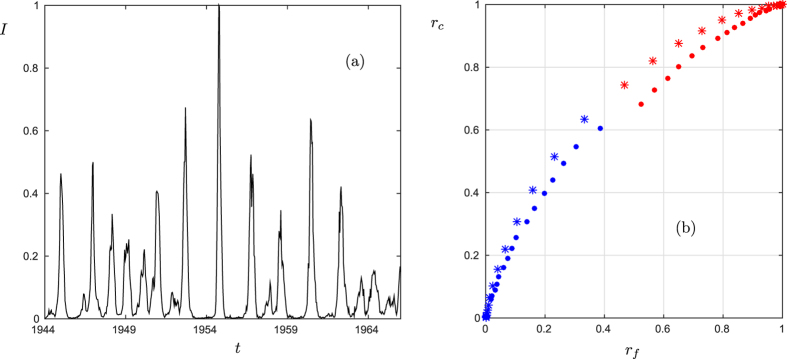
Measles epidemics in the UK between 1944 and 1966. (**a**) Typical time series of a city (here: Birmingham) for the infected population *I*; the series has been normalized by the maximum outbreak^37^,^38^. (**b**) ROC curves for a five (dots) and ten (stars) data point prediction averaged over all cities. The diagonal is shown as well. Blue corresponds to a precursor volume with *δ* > 0 and red to a precursor volume with *δ* < 0. A prediction time window of 5 months is indicated by ‘stars’ while a time window of 2.5 months is indicated by ‘dots’.
