# Supplementary material for: Early warning signs for saddle-escape transitions in complex networks
Source: Sci Rep. 2015 Aug 21;5:13190. doi: 10.1038/srep13190 (PMC4544003; doi:10.1038/srep13190)
Supplement: Supplementary Information [file srep13190-s1.pdf]

Supplementary information files for

„Early warning signs for saddle-escape transitions in complex networks“

by Christian Kuehn, Gerd Zschaler, Thilo Gross

Corresponding Author: Christian Kuehn, e-mail: ck274@cornell.edu

---

Supplementary Information

1 Metastability Near Saddle Points

Abundance of Saddles in Complex Systems Our main assumptions on complex networks is that steady states are "frequently" saddle points. In this section, we provide a mathematical justification for this assumption. Suppose we are given a large system of ODEs

$$x' = f(x) \tag{2}$$

for $x \in \mathbb{R}^n$ and $n \gg 1$. Assume x^* is a steady state (or equilibrium point, $f(x^*) = 0$) for (2). Denote the linearization (or Jacobian) $Df(x^*)$ by $A_n \in \mathbb{R}^{n\times n}$ which generically provides the local stability of x^* using the Hartman-Grobman Theorem[4]. Eigenvalues of A_n with negative/positive real parts correspond to stable/unstable eigendirections. Under the hypothesis that the system is complex, heterogeneous and its size is large it is reasonable to assume[17] that A_n is a random matrix with independent identically distributed (iid) entries given by a complex random variable z with mean zero and variance σ^2. Let $\{\lambda_i\}_{i=1}^n$ denote the eigenvalues of A_n and define the empirical spectral distribution μ_n of A_n by

$$\mu_n(s,t) := \frac{1}{n}\#\{k \leq n : \text{Re}(\lambda_k) \leq s \text{ and } \text{Im}(\lambda_k) \leq t\}.$$

where $\#$ denotes the cardinality of a set. Recently it has been proven (see[37] and references therein) that, under suitable boundedness assumptions on the moments of z, the circular law conjecture holds which states that μ_n converges to the uniform distribution over the unit disk

$$\mu_\infty(s,t) := \frac{1}{\pi}\text{mes}(\{w \in \mathbb{C} : |w| \leq 1, \text{Re}(w) \leq s \text{ and } \text{Im}(w) \leq t\})$$

where ’mes’ denotes Lebesgue measure. Hence, asymptotically as $n \to \infty$, if λ is an eigenvalue of A_n then

$$\mathbb{P}(\text{Re}(\lambda) < 0) = \frac{1}{2} = \mathbb{P}(\text{Re}(\lambda) > 0).$$

This implies that x^* is stable with probability $(1/2)^n$, completely unstable with probability $(1/2)^n$ and a saddle point with probability $1 - (1/2)^{n-1}$. Therefore, the probability that x^* is a saddle point tends to one as $n \to \infty$.

It is extremely important to note that the argument here is based upon certain mathematical assumptions to make it rigorous. However, it is strongly expected that if we *weaken* the assumptions in various ways, we still find saddle points frequently in high-dimensional systems. In fact, let us point out that the idea to characterize instability in large-scale systems using random matrix theory is well-known[38] but is still a topic of very recent interest[39]. However, previously one only had the semi-circular law available that required the symmetry of A_n or one had to rely on structured matrices, for example certain types of food webs[17]. These assumptions are usually too strong for complex dynamical networks, which can be highly heterogeneous and yield unstructured, non-symmetric ODEs. This makes the recent progress on proving the full circular law conjecture important in our context. As discussed above, one expects that the results can be generalized even further to include even larger classes of complex systems. Furthermore, note carefully that even if n is small, one may still have saddles, which may be relevant for the dynamics, i.e., the assumptions we make are *sufficient* to prove saddle existence with high probability but the assumptions may not be *necessary* to find saddles at all.

Residence Times Another main point of our argument is that the systems can spend a much longer time near saddle points than away from them. This leads to metastable behavior near saddle points. This can be illustrated with the simplest two-dimensional case given by the ODEs

$$\begin{aligned} x_1' &= \lambda_s x_1, \\ x_2' &= \lambda_u x_2, \end{aligned} \tag{3}$$

where $\lambda_s < 0 < \lambda_u$. Note carefully that it is justified to reduce the dimension of the system *after* the large dimensionality of the system has led to saddle points; the mathematically rigorous reduction just follows from center manifold theory [4]. The system (3) decouples with solution

$$x_1(t) = x_1(0)e^{\lambda_s t} \qquad \text{and} \qquad x_2(t) = x_2(0)e^{\lambda_u t}.$$

Suppose we start with some $x_1(0) = \kappa > 0$ and want to reach a small neighborhood of the origin with $x_1(T) = \delta \ll \kappa$. This takes a time $T = \lambda_s^{-1} \ln(\delta/\kappa)$. Viewing T as a function of κ shows that the time increases logarithmically. Therefore, a trajectory spends a much longer time near the equilibrium in comparison to the approach towards the equilibrium. Similarly, we can require a trajectory to start in a small neighborhood of the saddle with $x_2(0) = \delta > 0$ and end at $x_2(T) = \kappa > 0$. Then $T = \lambda_u^{-1} \ln(\kappa/\delta)$ and the same arguments apply to show that the initial time spend near the equilibrium is much longer than the escape time. Although we have only worked with a linear system (3), similar conclusions apply for the nonlinear case as long the passage near the hyperbolic saddle occurs sufficiently close to its stable and unstable manifolds[4].

2 Saddle Point Warning Signs

Basics Locally near a hyperbolic saddle point, which we can assume without loss of generality to be at $x = (0, 0, \ldots, 0) =: 0$, we can work with the linearization so that

$$x' = Ax \tag{4}$$

for some matrix $A \in \mathbb{R}^{n\times n}$ with eigenvalues $\lambda_i \in \mathbb{C}$ for $i \in \{1, 2, \ldots, n\}$ and associated eigenvectors v_i. We are going to assume that the eigenvalues are distinct which is generic within the space of matrices. Standard linear algebra gives a coordinate transformation $P : \mathbb{R}^n \to \mathbb{R}^n$, $x = Py$, so that

$$P^{-1}AP = B = \begin{pmatrix} B_1 & & \\ & \ddots & \\ & & B_k \end{pmatrix}$$

where the k matrices $B_1, \ldots, B_k$ are the usual Jordan blocks and P maps the standard basis vectors to the basis $\{v_i\}$. Since the eigenvalues are distinct we have $B_j \in \mathbb{R}$ or $B_j \in \mathbb{R}^{2\times 2}$. It is straightforward to observe that the escape near saddles is governed by the weakest stable and the strongest unstable directions. More precisely, we will only consider at most four eigenvalues $\lambda_s, \overline{\lambda_s}, \lambda_u, \overline{\lambda_u}$ where overbar denotes complex conjugation so that

$$\begin{aligned} &0 > \text{Re}(\lambda_s) > \text{Re}(\lambda_k), && \text{for all } k \neq s \text{ such that } 0 > \text{Re}(\lambda_k), \\ &0 < \text{Re}(\lambda_k) < \text{Re}(\lambda_u), && \text{for all } k \neq u \text{ such that } 0 < \text{Re}(\lambda_k). \end{aligned} \tag{5}$$

It is extremely important to highlight again the logic in the previous derivations: First, we start with a large-dimensional system, where it can be shown that saddle points are frequent. For each hyperbolic saddle point, there are many eigenvalues. However, for the dynamical approach or

departure of the saddle point, the dynamics is locally governed by the weakest stable and strongest unstable directions, i.e., stronger stable directions damp out very quickly, while weak unstable directions are generically dominated by the strongest unstable mode. Hence, we may develop a local theory for high-dimensional hyperbolic saddles by focusing on the leading directions in the stable and unstable manifolds, which are generically low-dimensional.

The Planar Saddle We start with the case $n = 2$ and $\lambda_{s,u} \in \mathbb{R}$. Setting $x = Py$ gives $y' = P^{-1}APy = By$ with solution $y(t) = y(0)e^{tB}$ or $x(t) = Py(t)$ so that

$$x(t) = y_1(0)e^{\lambda_s t}v_1 + y_2(0)e^{\lambda_u t}v_2.$$

for vectors $v_{1,2}$ that can be calculated explicitly. Since we want to approach the saddle point and stay near it for some significant amount of time we must have that $|y_2(0)| \neq 0$ is small so that a solution starts close to the stable manifold $W^s(0) = \{\rho v_1 : \rho \in \mathbb{R}\}$. Let $\|\cdot\|$ denote the usual Euclidean norm. Observe that the term $\|y_1(0)e^{\lambda_s t}v_1\| \to 0$ as $t \to \infty$ and, if $y_2(0) \neq 0$, $\|y_2(0)e^{\lambda_u t}v_2\| \to \infty$ as $t \to \infty$. Hence, initially $\|x(t)\|$ decreases exponentially until a unique minimum and then $\|x(t)\|$ increases exponentially. For times $t_j > 0$ such that $y_2(0)e^{\lambda_u t_j}$ is small it follows for some $t_2 > t_1 > 0$ that

$$\|x(t_2) - x(t_1)\| \approx |y_1(0)||e^{\lambda_s t_2} - e^{\lambda_s t_1}|\|v_1\| = |y_1(0)|\|v_1\||e^{\lambda_s t_1}(e^{\lambda_s (t_2 - t_1)} - 1)|$$

If $t_2 \gg t_1$ then $e^{\lambda_s(t_2-t_1)} \approx 0$ so that

$$\ln \|x(t_2) - x(t_1)\| \approx \lambda_s t_1 + k_1 \tag{6}$$

where $k_1 = \ln(|y_1(0)|\|v_1\|)$ is a constant that will not be of relevance here. Observe that (6) allows us to estimate λ_s from data. Then we can consider it a warning sign when the logarithm of the

distance between points starts to deviate from the linear fit (6). In the regime where $|y_1(0)|e^{\lambda_s t}\|v_1\|$ is small a similar procedure allows us to estimate the strongest unstable eigenvalues since then we find

$$\ln \|x(t_2) - x(t_1)\| \approx \lambda_u t_2 + k_2 \tag{7}$$

where $k_2 = \ln(|y_2(0)|\|v_2\|)$. From the knowledge of λ_u we can predict how rapidly $\|x(t)\|$ is expected to grow. In practice, we can estimate the eigenvalues $\lambda_{s,u}$ from a uni-variate coordinate time series $x_i(t)$ by looking at a fixed time point T and a set of K previous times $t_1 < t_2 < \cdots < T$ to compute

$$d_i(T) := \frac{1}{K}\sum_{k=1}^{K} \ln |x_i(T) - x_i(t_k)|. \tag{8}$$

Computing $d_i(T)$ for different times T gives that in different regimes (stable/unstable) we have $d_i(T) \sim \lambda_{u,s}T + K_2$ for some constant K_2.

Complex Eigenvalues For the case $n = 3$ we will again assume that (5) holds and that the complex conjugate eigenvalue pair has negative real part i.e. we consider $\lambda_s = a_s + ib_s$, $\overline{\lambda_s} = a_s - ib_s$, λ_u with associated real eigenvectors $v_{1,2,3}$. With $x(t) = Py(t)$ the general solution is

$$y(t) = \begin{pmatrix} e^{a_s t}[y_1(0)\cos(b_s t) + y_2(0)\sin(b_s t)] \\ e^{a_s t}[y_2(0)\cos(b_s t) - y_1(0)\sin(b_s t)] \\ y_3(0)e^{\lambda_u t} \end{pmatrix}.$$

Writing the solution for $x(t)$ in the basis of the eigenvectors v_i gives

$$\begin{aligned} x(t) &= e^{a_s t}[y_1(0)\cos(b_s t) + y_2(0)\sin(b_s t)]v_1 \\ &\quad + e^{a_s t}[y_2(0)\cos(b_s t) - y_1(0)\sin(b_s t)]v_2 + y_3(0)e^{\lambda_u t}v_3. \end{aligned}$$

As before, we are going to distinguish two regimes in the time domain, starting with the assumption that $\|y_3(0)e^{\lambda_u t}v_3\|$ is small which yields exponentially decaying oscillations in time series for each coordinate x_i. Let T be the time between successive maxima or minima then $b_s = 2\pi/T$. Furthermore, if $t_2 > t_1 > 0$ as previously and $t_2 - t_1 = T$ then

$$\|x(t_2) - x(t_1)\| \approx k_3|e^{a_s t_2} - e^{a_s t_1}|$$

for a positive constant k_3. The last equation can then be used to estimate a_s as shown in Section 2. The case of two complex conjugate eigenvalue pairs is similar and will not be discussed here.

Noisy Saddles An important question is to consider the influence of noise as natural systems, and in particular the measurement of natural systems, are often well-described by an underlying deterministic system with additional random fluctuations. Consider the standard one-dimensional Ornstein-Uhlenbeck (OU) $x = x(t)$ stochastic process generated by the stochastic differential equation (SDE)

$$\mathrm{d}x = \frac{1}{2}ax\ \mathrm{d}t + \sigma\ \mathrm{d}W \tag{9}$$

where $W = W(t)$ is a standard 1-dimensional Brownian motion and the equation is interpreted in the Itô-sense. It is well-known[40] that the solution to (9) and the resulting variance $V(t) = \text{Var}(x(t))$ can be calculated

$$V(t) = \frac{\sigma^2}{a}\left(e^{at} - 1\right).$$

For $a < 0$ it follows that $V(t) \to -\sigma^2/a$ as $t \to \infty$ and for $a > 0$ one gets $V_t \to \infty$ as $t \to \infty$. More precisely,

$$\ln(V(t)) = \ln\sigma^2 - \ln a + \ln\left(e^{at} - 1\right) \sim 2\ln\sigma - \ln a + at \qquad \text{as } t \to \infty. \tag{10}$$

Generalizing (9) to the simplest possible saddle point yields

$$\mathrm{d}x = Ax\;\mathrm{d}t + \sigma\;\mathrm{d}W \tag{11}$$

where $W = W(t)$ now denotes a standard 2-dimensional Brownian motion and A has two eigenvalues $\lambda_s < 0 < \lambda_u$. Then the same conclusion as before apply since the entries of the covariance matrix $C(t) = \text{Cov}(x(t))$ are generically linear combinations of two decoupled OU-processes, one stable with asymptotically constant variance for $a = \lambda_s$ and one with diverging variance for $a = \lambda_u$. Hence one could also attempt to use the scaling (10) to get an estimate for λ_u by considering a moving window analysis of the logarithm for the variance. This could be of particular interest in case the more straightforward logarithmic distance reduction method does not work. Most likely this will be the case only for very particular intermediate noise strengths. For small noise the estimator based on distances works quite well as shown in the main manuscript. However, if the noise is too large one never reaches a neighborhood of the saddle point with high-probability so that predictions become impossible anyway i.e. the events acquire a purely noise-induced character.

An Example from Epidemics Saddle points also appear frequently in many applications. Here we briefly illustrate the dynamics near saddles for a compartmental epidemic model[31,41]. The basic model is given by

$$\begin{aligned} S' &= -\phi I^2 S/T^2 - dS + \rho R + bT, \\ I' &= \phi I^2 S/T^2 - (d+\gamma)I, \\ R' &= \gamma I - (d+\rho)R, \end{aligned} \tag{12}$$

where S, I, R are the number of susceptible, infected and recovered individuals in the population (with $T := S+I+R$), d and b are per capita death and birth rates, γ is the per capita recovery rate,

and ρ is the per capita loss of immunity constant. ϕ is the main bifurcation parameter of the model and controls the interaction strength for the nonlinear incidence function I^2S. Assuming $d = b$ to keep the total population constant, introducing the new variables

$$s := S/T, \qquad i := I/T, \qquad r = R/T,$$

and using the constraint $1 = s + i + r$, one arrives at a two-dimensional ODE system[31]

$$\begin{aligned} i' &= -i[\phi i(1 - i - r) - (b + \gamma)], \\ r' &= \gamma i - (b + \rho)r. \end{aligned} \tag{13}$$

The system (13) has a number of different dynamical regimes depending upon the parameter values; for example, various bifurcations occur such as fold and Hopf bifurcations. Figure 5 shows a simulation of the dynamics for parameter values $b = 1$, $\rho = 0.132051$, $\phi = 81.88$, $\gamma = 1.4$ and initial condition $(i, r) = (0.2, 0.3)$. In the (i, r)-phase space plot in Figure 5(a) a saddle point $(i, r) = (i^*, r^*)$ has been marked as a dot. For the chosen initial condition, the trajectory approaches a neighbourhood of the saddle several times, where it only evolves slowly as discussed above. These long periods near the saddle are then interspersed with several short periods consisting of large epidemic outbreaks. The dynamics in this parameter regime is transient and will settle after a very long time to a stable sink equilibrium. However, before this occurs, the saddle dynamics plays the main role. Figure 5(b) shows a time series of the infected population density i corresponding to the trajectory from Figure 5(a).

It is important to note that the observed effect can also occur for the case when the saddle point lies precisely on the zero line. Indeed, if we consider a coordinate change $\tilde{i} = i - i^*$, then the

saddle point lies on the zero line $\{i = 0\}$ and we observe the same saddle escape phenomenon as above.

3 Cooperation Games on Networks

In this section we give a more detailed technical description of the snowdrift game network. The evolutionary game is defined between agents (nodes) that interact on an adaptive network via the links between them[22]. The number of nodes N and number of undirected links K is fixed. However, as described below, the adjacency matrix $A = (a_{ij})$ may change in time and interacts with the dynamics. This makes the system and adaptive, or co-evolutionary, network. An agent i interacts with an agent j at a given time step if there is a link between i and j. The interaction takes place via a game between the two nodes. In this game, an agent can have two possible strategies σ_i, cooperation C or defection D, which are the two dynamical states of the nodes. The payoff agent i receives from agent j via an interaction is modeled via the snowdrift game[23] with interaction matrix

$$M = \begin{pmatrix} b - c/2 & b - c \\ b & 0 \end{pmatrix}$$

where c represents the cost of cooperation and b the benefit. M_{11} represents cooperation of both agents, M_{22} defection of both agents and the off-diagonal entries correspond to the mixed cases where one agent tries to cooperate but the other agent defects. The total payoff π_i for node i is

$$\pi_i = \sum_{j:a_{ij}=1} M_{ij}.$$

The network is made adaptive by a probabilistic rule. After the game has been played, choose a link at random. With probability p re-wire this link and with probability $1-p$ one of the linked agents adopts the other agent's strategy. The two events of re-wiring and adaptation have to specified in more detail. Define the performance $\phi(\sigma)$ of a strategy $\sigma \in \{C, D\}$ as

$$\phi(\sigma) := \frac{1}{n_\sigma N} \sum_{i:\sigma_i=\sigma} \pi_i$$

where n_σ is the fraction of agents using strategy σ. If the strategy adoption event takes place agent j adopts the strategy of agent i with probability

$$f_\beta(i,j) = \left(1 + e^{-\beta[\phi(\sigma_i)-\phi(\sigma_j)]}\right)^{-1}$$

and i adopts j's strategy with probability $f_\beta(j,i) = 1 - f_\beta(i,j)$. For a re-wiring event of a link between i and j, delete it and select a random node k. Then the link between k and i is generated with probability $f_\alpha(i,j)$ and between k and j with probability $f_\alpha(j,i)$.

The main dynamical variables we are interested in are the fraction of cooperators and defectors n_C and n_D, as well as the link densities l_{CC}, l_{CD} and l_{DD}. Since the number of nodes and links is constant it suffices to restrict attention to a single node density and two link densities. Consider the parameter set

$$\alpha = 30, \quad \beta = 0.1, \quad b = 1, \quad c = 0.8, \quad N = 50000, \quad K = 500000$$

where the re-wiring rate p is the primary bifurcation parameter that is varied between $p = 0$ and $p = 1$. There are three main dynamical regimes, for small p the densities n_{CD} remain almost constant and there are just finite-size effect stochastic fluctuations. Note that for small p, the

network topology is very close to being static. Increasing p gives rise to a supercritical Hopf bifurcation to oscillations in a system, where the population and link densities between different types of agents are taken into account[22], i.e. the linearization at the near-homogeneous steady state has a pair of complex conjugate eigenvalues, which cross the imaginary axis at nonzero speed upon variation of p. This leads to small-scale deterministic oscillations, which grow in amplitude upon increasing p further. For large p the periodic dynamics approaches a near-homoclinic orbit with saddle-type escape dynamics and long periods of high cooperation values with n_C near 1. More precisely, the period of the oscillations increases and long times are spend near a saddle steady state and eventually the periodic orbit limits onto a homoclinic orbit, which means trajectories come extremely close to the saddle steady state. Note carefully that high values of p mean that the network topology can change very quickly, i.e. quickly in comparison to the changes of the dynamical states of the agents. This leads to a highly heterogeneous complex system, which can drastically change its entire topology and dynamical state; for a more detailed description of the dynamics we refer to[22].

4 Epidemics on Networks

In this section we provide a more detailed overview of the epidemiological network model discussed in the main text. The total number of nodes N and links L is assumed to be fixed. Nodes can be in either in a susceptible (S) or infected (I) state. Links between nodes represent potential transmission routes of the disease. Loops and double-links are not allowed. At each time step an infected node recovers with a probability (or recovery rate) r into a susceptible node. For every

SI-link the disease spreads with probability p so that the S node becomes an I node upon infection. This basic dynamics just represents the standard susceptible-infected-susceptible model[42]. In addition, susceptibles can try to avoid contact with infected and this is modeled via a probability w of re-wiring an SI-link. In this case, the susceptible node S cuts its link to a node I and establishes a new link to another susceptible node.

As before, we are mainly interested in the node and link densities and their trajectories. It is natural to view the parameters (r, p, w) as bifurcation parameters. We briefly describe the dynamics that have been found in[24]. It is observed that cluster formation and degree correlation depend on the re-wiring r, e.g. higher re-wiring rates lead to higher degree correlation. Furthermore, the main bifurcation point upon varying w is the epidemic threshold corresponding to a transcritical bifurcation. Hopf bifurcations, saddle-node bifurcations, oscillations and hysteresis can occur.

The re-wiring mechanism can be refined by introducing awareness of susceptibles to the disease. Let $\rho = i/N \in [0, 1]$ denote the infected fraction of the population. Now define $w = w_0\rho$ where w_0 is a fixed constant. In addition to the bifurcation phenomena observed previously, a homoclinic bifurcation is found upon varying p. The large-amplitude oscillations that occur near the homoclinic bifurcation are of interest for our study of saddle escapes in the main text.

The parameter values used for full network simulation are $p = 0.0058$, $r = 0.002$ and $w_0 = 0.6$ with an initial susceptible density $s_{t=0} = 0.98$. The total number of nodes was fixed to $N = 10^5$ and the total number of links to $L = 10^6$. These yield oscillations and their analysis using saddle-type escape dynamics gives the results shown in (Fig. 3).

5 Measles Data

The data set we use for testing our methods are measles epidemic data recorded in 60 UK cities from 1944 to 1966; the data has been downloaded from the website[43,44]. A detailed description and analysis of this data set is given in the two papers[27,28]. For each city the total number of cases has been reported biweekly. Depending on the size of the city, measles is expected to occur in endemic cycles (large cities) or in recurrent epidemics with local extinction. It is important to observe that either of those two phenomena depends on the fact that the virus is somewhere in the total host population. Therefore, a low infected density is generically expected to lie very close to the stable manifold of a saddle point when considered in a sufficiently large phase space.

An interesting aspect of the modeling of Grenfell et al.[27,28] is that, although their model is stochastic, they recognize that during the initial phase and throughout the epidemic, the underlying dynamical system seems to be behave deterministically. This is precisely the same behavior one can observe from a model such as the epidemiological adaptive-network model described in the last section.

6 ROC curves

Here, we briefly review the main idea of ROC (receiver operating characteristic), also called the ROC curve, and give the formal definitions for the general case. Denote the points in a given time

series by $I_j := I(t_j) \in \mathbb{R}$ for $j = 1, 2, \ldots$ and let Y_m be a binary random variable with

$$Y_m := \begin{cases} 1 & \text{a tipping/event occured at time } m \\ 0 & \text{no tipping/event occured at time } m, \end{cases}$$

i.e. Y_m just records whether a tipping point occured at time m or not. Y_m is taken as a random variable since we do not a priori when events occur. Consider some subset of previous observations $I_{k_1,k_2} := (I_{m-k_1}, I_{m-k_1-1}, \ldots, I_{m-k_2}) \in \mathbb{R}^{k_1-k_2+1}$ with $0 < k_2 < k_1$, where $k_1 - k_2 + 1$ is also referred to as the sliding window length or observational window length.

Next, one defines a precursory variable $X_m := \text{pre}(I_{k_1,k_2})$, where pre : $\mathbb{R}^{k_1-k_2+1} \to \mathbb{R}$ is a mapping, which computes out of the observations a scalar-valued precursor. Obviously the variable X_m can depend upon the choice of k_1, k_2 and on further parameters (this problem is currently being studied by the first author and several colleagues for the case of B-tipping). We give an alarm when $X_m > \delta$ for the event some $\delta \in \mathbb{R}$. The precursory variable enables us to calculate the rate of correct predictions as well as the rate of false positives

$$r_c = \frac{\#\text{correct predictions}}{\#\text{events/outbreaks}} \quad \text{and} \quad r_f = \frac{\#\text{false positives}}{\#\text{non-events}}.$$

Both rates will obviously depend upon k_1, k_2, δ and the dependence will be upon δ only if the window length is fixed. One may also write r_c and r_f by using aposterior probability density functions as[29]

$$r_c = \int_{\{X_m > \delta\}} \mathbb{P}(X_m | Y_m = 1), \qquad r_f = \int_{\{X_m > \delta\}} \mathbb{P}(X_m | Y_m = 0).$$

The ROC-curve is a plot of the rates in the (r_f, r_c)-plane for different values of the threshold δ; see (Fig. 4) for an example. There are several important standard observations about ROC curves.

Perfect prediction occurs if no false positive and all true events are detected. This implies that the ROC curve should consist just of the point $(r_f, r_c) = (0, 1)$. The point $(0, 0)$ in the lower-left corner of an ROC curve represents a value of δ that is so high that no alarm is given at any point while the point $(1, 1)$ at the upper right corner represents when alarms are given at every time step. The diagonal connecting $(0, 0)$ and $(1, 1)$ is precisely, where true positive rate equals the false positive rate, which is equivalent to making random guesses. A precursor with performance better than random guesses corresponds to a point in the upper triangle with $r_c > r_f$.

Supplementary Figure 1 Numerical simulation for the compartmental epidemic model (13). (a) Phase space plot with saddle point (green dot) and a trajectory segment (black curve) showing multiple epidemic outbreaks with passages near a saddle. (b) Time series for the infected population density i corresponding to the trajectory from (a). The i-value of the saddle point is indicated by a dashed green line. The long passages near the saddle between larger epidemic ourbreaks are clearly visible on this time scale.

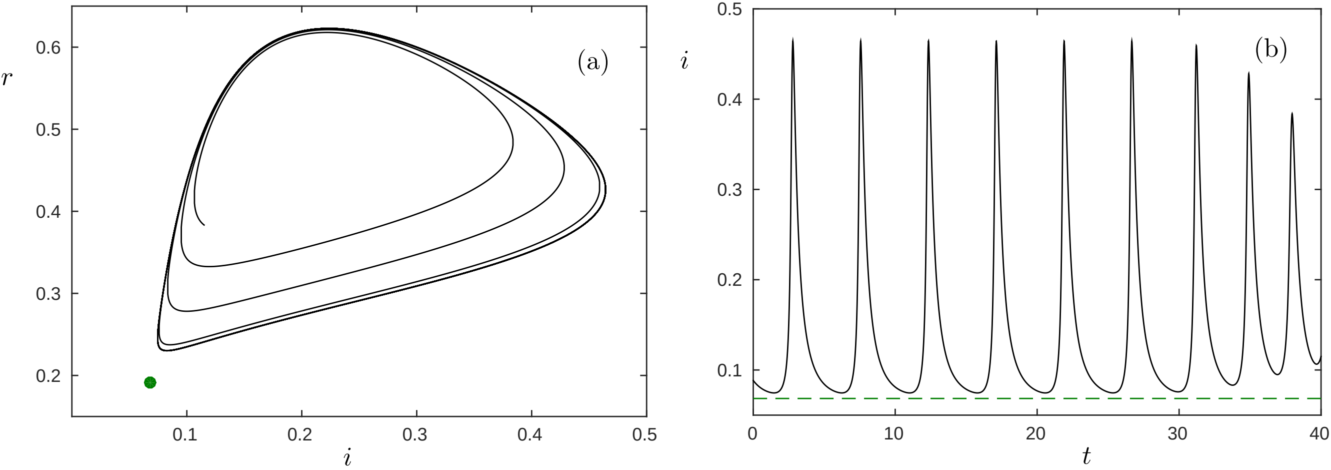

(a)
r
0.6
0.5
0.4
0.3
0.2
0.1
0.2
0.3
0.4
0.5
i
(b)
i
0.5
0.4
0.3
0.2
0.1
0
10
20
30
40
t
